# The Mark, the Thing, and the Object: On What Commands Repetition in Freud and Lacan

**DOI:** 10.3389/fpsyg.2017.02244

**Published:** 2017-12-22

**Authors:** Gertrudis Van de Vijver, Ariane Bazan, Sandrine Detandt

**Affiliations:** ^1^Centre for the History of Philosophy and Continental Philosophy, Ghent University, Ghent, Belgium; ^2^Service de Psychologie Clinique et Différentielle, Research Center for Clinical Psychology, Psychopathology and Psychosomatics, Université Libre de Bruxelles, Brussels, Belgium

**Keywords:** Freud, Lacan, repetition compulsion, jouissance, *fort-da*, beyond the pleasure principle, representation, dopamine

## Abstract

In *Logique du Fantasme*, Lacan argues that the compulsion to repeat does not obey the same discharge logic as homeostatic processes. Repetition installs a realm that is categorically different from the one related to homeostatic pleasure seeking, a properly subjective one, one in which the mark “stands for,” “takes the place of,” what we have ventured to call “an event,” and what only in the movement of return, in what Lacan calls a “thinking of repetition,” confirms and ever reconfirms this point of no return, which is also a qualitative cut and a structural loss. The kind of “standing for” Lacan intends here with the concept of repetition is certainly not something like an image or a faithful description. No, what Lacan wishes to stress is that this mark is situated at another level, at another place, it is “*entstellt*,” and as such, it is punctually impinging upon the bodily dynamics without rendering the event, without having an external meta-point of view, but cutting across registers according to a logics that is not the homeostatic memory logics. This paper elaborates on this distinction on the basis of a confrontation with what Freud says about the pleasure principle and its beyond in *Beyond the Pleasure Principle*, and also takes inspiration from Freud’s *Project for a Scientific Psychology.* We argue that Lacan’s theory of enjoyment takes up and generalizes what Freud was after in *Beyond the Pleasure Principle* with the *Wiederholungszwang*, and pushes Freud’s thoughts to a more articulated point: to the point where a subject is considered to speak only when it has allowed the other, through discourse, to have impacted and cut into his bodily pleasure dynamics.

## Introduction

It is well into his life as a practicing psychoanalyst that Freud wished to come to a firmer theoretical grounding of the clinical observation that people do not necessarily want to get rid of their suffering or their symptoms. Against therapeutic efforts of all kinds, people time and again *repeat*, even *cannot not but repeat*, what makes them suffer. This is what Freud means by *Wiederholungszwang*, the compulsion to repeat, situated, so he says, beyond the pleasure principle. In the text with the same name, *Beyond the Pleasure Principle* (Freud, 1920/1955), he explores the theoretical underpinnings of this clinical phenomenon. The idea he defends here is that the principle of repetition, and not the pleasure principle, is the most basic module of mental life, grounded in the drives. Freud is bold in his clinical affirmation – yes, the basic module of mental life is the compulsion to repeat, and not the pleasure principle^1^ – but he clearly does struggle to articulate the *Wiederholungszwang* in relation to the pleasure principle.

In *Logique du Fantasme*, Lacan (1966–1967/2017) invites us to consider that *Beyond the Pleasure Principle* constitutes a “conceptual intrusion” in Freud’s work. He insists: “Do we really measure what is at stake here?” To him, the *Wiederholungszwang*, articulated in terms of *jouissance*, enjoyment, constitutes a genuine break with the pleasure principle, a contradiction even with what Freud would have thought until then to be the module of the functioning of the mental system, namely homeostasis, that holds that living substances always seek the state of minor tension. To Lacan, there is no doubt about the fact that the pleasure principle reissues homeostasis for mental life: the mental system, in as far as it is ruled by the pleasure principle, “echoes,” “repeats,” “redoubles” organic, homeostatic requirements.^2^

In this paper, we propose to clarify what is at stake in Lacan’s diagnosis of a “conceptual intrusion” in Freud’s text. We argue that Lacan’s theory of the signifier and of enjoyment basically takes up and generalizes what Freud was after in *Beyond the Pleasure Principle* with the *Wiederholungszwang*, and that, notwithstanding the overt differences in style – Freud being more versed into biological metaphors and concepts, Lacan more into logical and topological formalizations – it is not the case that Lacan’s theory of the signifier with its focus on formalization is far removed from the apparently more bodily concerns of Freud. On the contrary, Lacan pushes Freud consequently to the point where the act of speaking itself is shown to involve an ineliminable place of the speaking other, while also having a subversive impact on what constitutes the homeostatic bodily pleasure dynamics.

This paper has two parts. In the first part, we explain what Lacan means with the idea that we are thinking with the object. This is important to come to clarity about his account of the signifier as participating in the dynamics of pleasure and repetition. To this end, we draw on Freud’s clinically interpreted anecdote of *fort-da*, and on Lacan’s re-interpretation of it in his seminars *The Four Fundamental Concepts of Psychoanalysis* (Lacan, 1963–1964/1973) and *La Logique du Fantasme* (Lacan, 1966–1967/2017). In the second part, we clarify and critically discuss how the use of signifiers introduces a radical cut organized around the limit points of the pleasure principle. Here, we depart from the distinction Freud introduces in his *Project for a Scientific Psychology* between understanding and judging, having it correspond, respectively, with the realm of representational, grasping bodily movements and the functioning of the mark, seen as a precursor and initiator of the properly subjective realm, with, between both, a relation of fundamental contingency or arbitrariness that serves as the ground for the compulsion to repeat.

## To Think With the Object: Freud and Lacan Interpreting the Children’s Game “*fort-da*”

In *Beyond the Pleasure Principle*, Freud re-discusses the *fort-da* children’s game^3^ to make it clear that the compulsion to repeat is not just to be equated with the repetition of painful events. Both the compulsion to repeat and the repetitive children’s games are related to the dynamics of excitations and their discharge, that is, to the pleasure dynamics. But they are so in a different way.

As early as Freud (1895/1966), proposes in his *Project for a Scientific Psychology* that homeostasis, the process that seeks the state of minor tension, is the default mode of mental functioning. This means that the mental system seeks in the first place to get rid of tension, that is, it is after the restauration of a previous state of less tension. Freud acknowledges of course that there is no mental functioning on that basis alone, because the mental apparatus also needs to be able to retain tension for a sufficient timespan, and it needs to be able to do this in organized ways, otherwise there would be no way of acting effectively in the surrounding world.^4^ The reality principle is what captures this requirement of retaining tension in order to adequately act and maintain oneself in the world. Both the pleasure principle and the reality principle, however, are eventually seeking a decrease in tension.^5^

The question is, of course, how much tension the mental system will or should be capable of retaining, and why or how it will do so. This matter is at the core of the Freudian distinction between the pleasure principle and the compulsion to repeat. In discussing the *fort-da* game, Freud stresses that the child, by staging the presence and the disappearance of his toys, compensates for the anxiety and the pain that the absence of the mother is provoking in him. The game repeats the supposedly painful event, which suggests that there is no immediate relief of tension involved. Freud, however, does not hesitate to say that the indemnification at stake involves a direct benefit. It concerns a different kind of pleasure though, one stemming from another source (Freud, 1920/1955, p. 17). The difference, so Freud explains, has to do with the fact that the child succeeds in being less passively subjected to what he experiences, has found ways to actively master, that is, to bind excitations through the throwing away and pulling back of his bobbin and the repetitive “o-a,” *fort-da*. In this way, discharge is enabled, while a distance is created with drive satisfaction. The use of “o-a” – that Lacan refers to as signs, marks – is what makes the mastering drive independent of whether the memory was itself pleasurable or not (*Ibidem*, pp. 16–17).^6^ However, even if the child’s mental functioning is independent of the initial objects of satisfaction, even if the use of signifiers installs the pleasure dynamics at another level, at another place, there is in Freud’s viewpoint on mental life still an “echoing,” “repeating,” “redoubling,” as Lacan states, of organic, homeostatic requirements. The module of mental life is homeostasis, that is, the pleasure principle.

Lacan’s (1963–1964/1973, p. 60) comments on the *fort-da* experiment in *The Four Fundamental Concepts of Psychoanalysis* are revealing for the questions that concern us here. Lacan is not siding with Freud’s clinically based distinction between the repetition at stake in the children’s game – still situated under the heading of the pleasure principle, albeit distinguished from mere drive satisfaction – and the “real” repetition he wishes to consider as lying beyond the pleasure principle, i.e., the compulsion to repeat. On the contrary, he considers the *fort-da* game as an instantiation of what constitutes mental life at heart, ruled, that is, by repetition, and, therefore, not ruled by a pleasure principle that echoes homeostasis. In this sense, repetition, also coined as enjoyment by Lacan, is in his view not “natural,” not obeying to what instincts or needs command in terms of pleasurable discharge.^7^ To Lacan, what counts in repetition, and what to him is illustrated in the *fort-da* game, is the attachment to that which stays the same, namely, the signifiers, “o-a,” *fort-da*, endlessly repeated.^8^ That the repetitive use of “o-a” takes place in an apparently signifying relation to a multiplicity of toys, in a variety of situations, is not what counts in the first place – it rather risks to distract us from its genuine significance. As a matter of fact, to consider the *fort-da* as a stamp of some or other event – the absence of the mother, for one, as Freud suggests – is too quickly complicit with a semantic-representational account and thereby misses the real point.^9^ According to Lacan, following here Wallon, the child is vigilant for what it experiences as a lack exactly next to him, in his vicinity, not where the mother left the room and where he could expect her to come back. In other words, the child does not constitute its mental life on the basis of, for instance, a “representation” referring to the mother leaving the room, expecting that she will come back at some point through the same door.^10^ It is in the vicinity where the lack makes itself *directly felt* that the play with the bobbin and the utterance of “o-a” take place. As the bobbin, the “o-a” is actually the little thing that is detachable from him while being still retained, the little thing on the basis of which the infant explores and expands his universe in a movement of self-mutilation – throwing the thing, part of his own movements, away, and thereby bridging the abyss created by the absence of what was in his vicinity a moment before “It is with his object that the child jumps over the borders of his territory changed in wells and that he begins the incantation” (Lacan, 1963–1964/1973, p. 60, our translation).^11^ The little subject of the *fort-da*, successfully finding discharge through the act of repetitively pronouncing “o-a,” is *in* the repetitive movements he initiates with his bobbin and covers with “o-a”.

So, in sum, Freud grounds the pleasure principle in the possibility of discharge, and quite logically considers the child’s game as a successful kind of discharge. What to him lies beyond the pleasure principle, has to do with those occasions where discharge appears to be problematic or radically impossible, such as in traumatic neuroses or in the phenomena of negative transference. To Lacan, however, this clinically observed distinction risks to miss the essential point, namely that in the signifying procedures whereby the child, or any speaking being for that matter, deals with absence and presence, there is a structural loss, a structural impossibility that inescapably emerges with the use of signifiers, with the use of “o-a.” This structural loss is not disconnected from the issue of discharge, and thus of pleasure, but does initiate another domain, obeying a different logics, a logics ruled by repetition. In order to make this clear, we have to explain how the use of the first signifier is connected to the pleasure dynamics, or rather, how it cuts with that dynamics and how it gives rise to the functioning of a new domain. To that end, we need to reconstruct and articulate in more detail how the child is caught into his movements, and how he finds an orientation on that basis.

## The Pleasure Principle and Beyond

### Understanding and Judging

We know that pleasure is seen by Freud as discharge of tension; it is a temporary, floating, and partial suspension of displeasure.^12^ Tension – displeasure, if not trauma – constitutes the background against which pleasure has to be thought. We also know that as long as we live, there is, structurally, the encounter with unpleasantly high levels of tension (Freud, 1895/1963, 1895/1966; Lacan, 1963–1964/1981). Within this setting, the first air entering the respiratory system, the first milk entering the digestive system, can likely be called traumatic experiences. What exactly is at stake in these experiences?

What Freud writes in his *Project for a scientific psychology* is relevant here. Freud makes a distinction between understanding and judging, that he grounds in the idea that the complex of what surrounds the child, the fellow human being in the first place, falls apart into two components, one which “makes an impression by its constant structure and stays together as a *Thing*, while the other can be *understood* by the activity of memory – that is, can be traced back to information from [the subject’s] own body” (Freud, 1895/1966, p. 331, italics original). So, to *understand*, is to find relief in and through the proper bodily movements, that is, to succeed in grasping something (*com-prehensio*), so that, by one’s own means or not, an effective handle is found on the basis of which discharge becomes possible. To *judge*, on the other hand, refers to something that resists understanding, a thing that for that reason “stays together as a Thing” and impresses by its constant structure, and that is to be covered and bridged by other means, with what Freud refers to as traits or marks (*Züge)*. To Freud, that is what judgment does: it corresponds to the impossibility of finding adequate movements that would lead to a grasp of the complex (understanding), and constitutes a cut with it by approaching the complex through a trait, a mark. A judgment is thereby “*entstellt*” with regard to understanding, it installs another realm, another domain.^13^

We propose to apply this schema to the dialectics between pleasure and displeasure and to what lies beyond. The potentiality of this move is twofold: on the one hand, it contributes to anchoring the functioning of the mark into the dynamics of pleasure and displeasure, approaching it, so to speak, “from below,” and, on the other hand, it allows to ground the (f)act of speaking in the obstacles and the impossibilities the human being encounters specifically in and through the motor patterns and their potentiality to lead to discharge. Let us return to our examples, the entering of milk in particular.

### The Thing, the Mark, and Primary Judgment

What happens in feeding, is that the child, most commonly, finds by itself the voluntary sucking movements that will contribute to the feeding.^14^ The act is what is first, with its motivational point – from where, why, for what reason it is undertaken – left unfathomable from within the system that undertakes it.^15^ As a consequence of the sucking movement, milk enters the system. That is, for the system, a surprise. That the movement of sucking, undertaken, so to speak, “out of nowhere,” would lead to milk entering, was not foreseen and could not be foreseen. The first milk that enters the system comes as a surprise, and cannot but come as a surprise; it constitutes an event: the milk is an external, *a priori* hostile element entering the system. However proximate the entering of milk with the sucking movements is, both are, for the system concerned, disconnected, in the sense that there is nothing in the act of sucking that is connected with milk: their relation is contingent. Also the fact that the sucking brings a certain relief simply related to the sucking itself, is initially disconnected from the milk and does not diminish the surprising effect of the latter. The event of milk entering the system for the first time is inscribed as a mark, but it is not understood – the milk “stays together as a Thing.” Our hypothesis is therefore that what is marked is first and foremost the event itself: the mark is the point, the punctual point expressing and inscribing the bodily surprise.^16^ The mark corresponds, in our view, to what Freud called the “*Triebrepräsentanz*,”^17^ that is, the point where the subject allowed for the fact of “being taken by surprise” and that opens the possibility of returning to that point. We consider this “being taken by the event in the form of a mark” as the first step in the process of subjective positioning, the first or primary judgment, corresponding to what Freud calls “*Bejahung*,” or what with Lacan becomes “*Bejahung pure, primitive*” (Lacan, 1955–1956/1981, p. 95) or “*Bejahung primaire*” (Lacan, 1966, p. 387). It does involve the position of the subject, albeit in a very preliminary and inviting sense: the fact that the trait was inscribed as a mark of the event, witnesses to a subjective choice – the little human let himself be surprised by the milk entering, it could as well have chosen *not* to drink. It therefore opens subjectivation as a task, an agenda.^18^ However, it is important to note that this logical time of being struck by surprise, is not exclusive to human beings, but, as we will explain further on, has to be supposed in vertebrates in general too.

What happens then with the undertaken movement that has made the entering of milk possible, and what about the relief to which it eventually contributed, or not? What role does it play in this “preliminary subjectivation”? Clearly, this movement, or cluster of movements, is of no help in *understanding* the event, but, being proximate, it gets linked to it, contingently but no less firmly. It is this link, inherently contingent but factually proximate, that, in our view, lies the ground for the further articulation of subjectivity, and of which Lacan will say that it is the ground for repetition.

What we propose here is that the adjacent movement, being only contingently linked to the ungraspable Thing of which the subject is factually experiencing the effects of surprise, this adjacent movement indicates and covers, “stands for,” the ungraspable Thing. Freud himself is speaking of *Vorstellungsrepräsentanz*, a term that caused a huge confusion among psychoanalysts and scholars.^19^ Freud sometimes identifies the *Triebrepräsentanz* with the *Vorstellungsrepräsentanz*, and there is something understandable at this. We would be inclined to say that the first adjacent movement, contingently sticking to the mark, so to speak, also already belongs to another realm, namely the realm of movements that can become representational. The adjacent movement thus carries both sides: it refers to the *Repräsentanz*, with the mark taking the place of the Thing and having the potentiality to elicit *Vorstellungen*, representations – these two different logical moments reflect Freud’s inaugural distinction between judgment at the one hand and comprehension at the other.

Beyond that first “sticky moment” where the *Repräsentanz* remains a pure potentiality – which is, actually, a logical moment, not a genetically identifiable moment^20^ – a call for other types of movements is launched, intentionally directed grasping movements this time, that are effectuated as a *return* to what escaped comprehension. Indeed, the first sucking is a sucking to discharge the sucking tension, but if it is followed by the event of the milk coming in^21^, and if it is thus given the weight of the mark, the subject can choose to have the next sucking as an intentionally directed movement, to grasp – i.e., to get – the milk. In this way, the *Repräsentanz* is what elicits representational – i.e., mental – activity. This subjective representational work is a work of understanding, of com-prehension, or at least, it is an attempt to understand, to grasp. This work is constituted on the basis of the marked adjacent movements – marks that are, as we have argued, the marks of a non-understanding, of a limit to understanding, that is, a limit to the possibility of grasping something, a limit to making that something (the milk entering as a surprise) into an object.

Very much in line with this, Lacan will consider the mark as the first signifier, S1, corresponding to Freud’s unitary trait, the “*Einzige Zug*,” the symbolic mark that constitutes an event by indicating a cut with the level of what is being marked (Lacan, 1966–1967/2017, p. 135). The S1 enables the primary judgment that has the form of an affirmation (*Bejahung*): it marks *that* there was an event that struck the body. However, to Lacan, the S1 has to be called symbolic already: in order to be called a mark at all, it intrinsically demands to be deployed and ever re-deployed through the articulation of representations that engage with other signifiers.^22^ If the mark would not have elicited the subject to a return, it would not be an *Einzige Zug*, an S1. S2 then stands for the chain of signifiers that aim at a return to the first signifier in an attempt to grasp or understand the initial moment of surprise, and in this sense corresponds to the representational activity which Freud refers to as the *com-prehensio*. Lacan speaks here of a “thinking of return,” a “thinking of repetition” (Lacan, 1966–1967/2017, p. 135).^23^ The articulation of this representational realm, its structuring, is properly symbolic, constituted of representations, but it is, meanwhile, very much anchored in the body, determined by what is, along a subjective history, being “accepted and inscribed” as a mark and what is contingently adjacent to it as an undertaken movement. In other words, what initiates the repetition of these actions is not the possible reward or relief they might bring about. What causes repetition is the fact that the action is being linked to the event, the event being constituted by surprise. Note that the marking, with an adjacent movement being contingently linked up with it, is *independent from* whether the event was painful (first air coming into the lungs) or rewarding (first milk entering the mouth cavity). In other words, the fact that milk enters the digestive system and eventually brings relief is *secondary* to the effect of the event *as such* – and it is the latter, not the former, that induces the repetition.^24^

What has to be further elaborated, therefore, is what this movement of “thinking of return” exactly involves, how it is marking specifically the human being as a speaking being, with representations becoming genuinely signifiers, and what, if anything, constitutes, in this context, the difference with the compulsion to repeat. In order to further unfold this, we need to turn to the status of the object, its relation to the possibility and the meaning of discharge, as well as to the role of the fellow human being in this fabric of pleasure and enjoyment.

### The Signifier Inscribed in a Basic Non-attunement of Actions and Needs: The Role of the Other

Let us return once more to the question of what happens in the deployment of directed actions by the subject, knowing that it must have been historically struck by the event, accompanied by the experience of the adjacent movement contingently linked to it. We know that all vertebrates capable of action have to cope with an initial non-attunement of actions and needs. The reason for this is structural. Vertebrates are characterized by a double body: an inner, invertebrate sack-like body with all the big vegetative systems (respiration, digestion, excretion, etc.) and a “newly invented” outer body constituted by a skeleton and striated muscles (see Bazan, 2007). While needs arise in the inner, invertebrate body, the specific actions for the satisfaction of these needs are outer body actions. The structural non-attunement between actions and needs resides in the fact that it is not *a priori* clear what outer body action could constitute a response to what the inner body needs. Even if this gap is less prominent in most vertebrates as compared to humans (e.g., little horses get on their feet and move toward the mother nipple in the span of hours after birth), the idea is nevertheless that, even in animals, this instinct-encouraged movement has to be sanctioned by a mark (a dopamine-release) to be registered as a movement with a high potential for repetition, and that therefore, independently of instincts, the body registers the history of (contingent) events. Once a specific action has been linked to an event, what drives to repeat this action, is disconnected from the drive satisfaction itself. Indeed, the relief caused by the satisfaction of an internal body need is only contingently connected to what the external body succeeded to develop as an action. *The relief, as the tension itself, is a serendipitous addendum, a by-product, important for survival, but not determinative for what drives repetitive behavior*. No matter what the outcome, the child will not stop the endless repetition, the sucking, or, as Freud stressed, the endless uttering of “o-o-o” and then “a-a-a.” From the moment the child accepted and marked the event, it is driven by the repetition compulsion to grasp. So, *it is from within the repetition compulsion that relief of tension becomes possible – it is not the relief of tension that is the ground for repetition*.

It is often said, from within a psychoanalytical setting, that what drives the human being is not the satisfaction of needs. We agree with this. In line with what we elaborated in relation to higher vertebrates, however, we consider that the structural non-attunement of needs and actions holds in the same way for human beings, and that it is intrinsically related to the way in which vertebrate bodies are constituted. This allows us now to address the question of the specificity of the human being, as a speaking being, from a slightly different angle. It is true, indeed, as Freud already highlighted in his *Project for a Scientific Psychology*, that the human child is born in a configuration of helplessness, which implies that the fellow human being plays a role that is structurally of the utmost importance in the constitution of his subjective world. Let us return to our example of the milk. Up until this point we have brought the scenario as if what is crucially at stake for the child is the sucking. However, due to his helplessness, the repetitive action of the sucking is, per force, supplemented by other actions, e.g., crying, vocalizations, that contingently, but crucially, contribute to realize the conditions within which relief becomes possible. Again, what drives the child to act and repeat its actions is not the possibility of relief in itself, it is the attempt to grasp what initially escaped, namely the surprising event. And in this grasping attempt, the other, as a speaking being, is once more an ineliminable factor. Indeed, a bunch of contingent movements, situated primarily in the realm of vocalizations, that, by surprise, out of nowhere, made a difference (i.e., brought the mother, the milk, relief), doubles, in a far more whimsical fashion, the logically first contingency of the sucking movement. Indeed, a mother, with far more fierceness than, e.g., milk, resists objectification, stays together as a Thing. More correctly, it is to the extent that the other “stays together as a Thing” and resists understanding, that the child is launched, here again, for an endless ‘thinking of return,’ a re-elaboration of his first vocalizations, in an attempt to grasp after all that which entered his system as a surprising event and with regard to which it did not succeed in articulating the appropriate adequate actions.

We therefore agree with Lacan when he states that the child thinks with his object, which means that he subjectivizes through the handlings with his object. We also agree with him (cf. his reading of “o-a”), that the use of signifiers, and the linguistic, signifying practices at large have to be understood along the same lines: a signifier is handled as an object, *the* object perhaps, on the basis of which the child explores and expands his subjective universe. We have explained in the previous part how these handlings are articulated in terms of a failure in grasping the Thing, how the marks in a sense “take over,” or at least initiate a new realm of being, the realm properly constituted by signifiers (which is the Other, in Lacan’s terms). We remind here that the signifier is a form, a motor-pattern, only contingently linked to what brings discharge – indeed, it marks precisely what was not understood and could not be brought back to memories of the proper body.

Repetition or enjoyment, a “thinking of return,” as Lacan calls it, is therefore, for the human being, intrinsically bound up with the nature of the signifier. The child, in the same movement of adopting the signifier that is offered to him as a formal potentiality by the other, inscribes himself in a universe of vocalizations where it is structurally impossible to grasp the Thing. It is from there on condemned to run after the Thing, to commemorate what can be called, perhaps, a moment of exquisite subjectivity – the structurally escaping moment of having been struck by surprise. By structurally missing this point because of the fact that the Thing is situated at another level and cannot be brought back to bodily understanding, the subject endlessly, repetitively, runs after “the facts”: it repeats the marks in themselves, and strives for understanding after all, attempts to make the Thing into an object, to bring it back to proper and directed body movements that bring discharge. Both realms, however much intertwined they are, are disconnected realms, only contingently bound up. As we saw with the “fort-da” game, the child produces the first signifiers “out of nowhere,” or at least, these signifiers cannot be grounded in the distinctions between his toys, between the mother or the father being absent or present. There is no way back from signifiers to meanings^25^: the relation between form and content is neither innately, nor naturalistically grounded. As we saw with the first mark, the *Einzige Zug*: signifiers emerge at the point where a “naturalistic” grounding – an adequate grasping of the object, leading to discharge – reaches a limit. Or perhaps more correctly: the use of the signifier indicates that a limit was reached, indicates that the bodily movements were inadequate.

What both Freud and Lacan note in relation to this repetitive dynamics, a thing that follows logically here, but needs to be stressed time and again, is that the action does not at all need to be adequate to be repeated. This is quite generally what clinical experience confirms: it may be certified that praying or singing does not stop the earthquake – it is nevertheless repeated. The child’s repetitive “o-a” does not impact on the leaving and the return of the mother, but it is joyfully and victoriously repeated nevertheless. We have defended elsewhere (Bazan et al., 2016) that even if the act is not adequate in grasping the thing, it still is better than sideration or bewilderment; it is the execution in itself that brings relief. Freud will say it is relief “from another source”; Lacan will consider that it radically concerns another domain, the domain of signifiers to which the child finds entrance. And in this domain, the other, the Other, occupies an ineliminable place: without the other/the Other, there would be no “significance,” no functioning of the signifiers. It is a space where contingency, or rather, arbitrariness between form and content reigns: the exchanges between the child and the other, in as far as they are based in signifier exchanges, are firstly formal exchanges, content being realized in the historical interweaving between those forms, the adjacent bodily movements and the web of directed and intentional actions deployed in their wake.

### The Object as a Coherent Motor Package and the Experience of Satisfaction

What is there to say then about the object? We would be inclined to consider an object as a coherent motor package, a bounded set of grasp movements that opens the possibility of discharge. We already said that the *Repräsentanz*^26^ “stands for” the event: it might be considered as a crystallization of movement parameters into a solidary whole. It is different from what philosophers are traditionally inclined to call an object, though, as it is a contingent whole of movements *arbitrarily* cut out of a sequence and has no intentional directedness. However, as argued, the motor pattern has the potentiality to launch for a return under the form of directed actions. These actions produce what we would call in the proper sense “mental” representations of what first intruded the system and stayed together as a *Thing*. It are then these “mental representations” that, when executed, can lead to discharge, however partial and temporary that is, and which can, in our view, be genuinely called *objects.* In other words, the mental, representational inscription amounts to an objectification. As this is likely to be the most delicate point of our argumentation, we dare to insist. Firstly, what we call an object or a representation is first and foremost a motor pattern, a motor intention, as Jeannerod calls it (Jeannerod, 1994; Bazan, 2007): it is the motor form within which something *can* be grasped. To address the question of the object, is therefore in the first place to ask for the formal arrangement of the space of possible motor patterns. What serves as a filling up in that space – content, meaning, … – is secondary, and does not inform about the arrangement of the space itself. In other words, questions about representations corresponding more or less adequately to some or other object out there, are missing the point. A representation *is* an object, *is* a motor pattern, and the articulation of the space of motor patterns, being a constraining space, *is* at the meantime the enabling condition for what counts as an object: the constraint *is* the possibility.^27^

This account of the object enables us to address (i) the typically Lacanian idea of the object as bound up with a structural loss – the fact of launching comprehending grasp movements is indicative of, rests on, a step being missed, as we have shown, the step corresponding to a non-understanding, covered by the mark – with the object *a* that theoretically indicates this ever missed object, (ii) the issue of objective reality, that here refers to successful grasping movements, i.e., movements leading to discharge. Clearly, the issue of discharge is crucial in the constitution of the object. As a matter of fact, only that which *can give rise* to discharge has a chance to lead to objectification. Along these lines, Lacan states that it is impossible to understand what an object is without the dimension of satisfaction, a thing that, to him, largely escaped the philosophical tradition.^28^ There is, however, a potential confusion in the way in which Lacan uses the term satisfaction, at least in as far as we take it to refer to the satisfaction of needs. We have indeed argued that there is a contingent, arbitrary relation between what brings satisfaction of needs and what is constituted as an object through motor-patterns. We have also argued that object-constitution happens on grounds radically cut from what brings satisfaction of needs. We are indeed speaking of another level, of pleasure “from another source” as Freud states, of objects of enjoyment, as Lacan calls it. In other words, *objects of pleasure are not situated at the level of the satisfaction of needs*^29^! We propose therefore to reserve the term satisfaction for the level of needs, to talk of objects of pleasure when we are aiming at the object constitution that is related to what brings relief, diminution of tension, and of enjoyment to indicate the impossibility of relief that the subject is desperately holding on to.

It is in relation to this that the exchanges with the fellow human being have to be investigated. Of course, the fellow human being is essential for what is to be called the constitution of objects of pleasure. In providing for the essential means of discharge – carrying out the specific acts – the other structurally intervenes in the temporality of excitation and discharge of the child, co-determines the identification of what counts as an *object* of pleasure, and in this way also co-determines what lies beyond in terms of enjoyment. That this has its implications for what counts as an experience of satisfaction is evident, but the important thing to note is that it is not the satisfaction that determines the constitution of the objects of pleasure or of enjoyment. Rather, the satisfaction of the need, in this new scheme, is over-written or replaced by the possibility of discharge through grasping movements leading to objectification, a possibility that was opened up as a return to the marked event covering that which stayed together as a Thing. The space of satisfaction of needs is thereby subverted into a space whereby the subject is endlessly and repetitively demanding to be recognized at another “level,” the one of subjectivity, expecting from the other to tell him the answer, that is, to bring (to be), for him, the object of relief.

## Conclusion

In *Beyond the Pleasure Principle*, Freud seeks to describe and articulate the functioning of the psychic apparatus *in situ*, that is, anchored in the ways in which human beings sense, move and act. In discussing the issue of *Wiederholungszwang*, Freud, here perhaps more than anywhere else, starts from the clinical observation of quantities of excitation of which it is not easy, not possible even, for the subject to get rid. That is where to him the disjunction between pleasure and repetition finds entrance: at the point that cannot be silenced through understanding, the point where pleasure, the possibility of decreasing tension, has come to a limit, the point that in its insistence searches other ways out. Freud’s overt biological phrasing is certainly not a matter of looking to ground the psychical in the biological; it is a matter of cutting the psychical at the correct joints. And this cutting cannot but start from the embarrassment in relation to the body, that is, from the moments and the points where something does not obey the logics of pleasure and lies beyond it as a compulsion to repeat.

In line with this viewpoint, and taking up Lacan’s revisiting of it in terms of enjoyment, we have argued (i) that the insistence with which subjects repeat is to be grafted upon the structural disconnectedness between what articulates behavior and what satisfies needs, (ii) that this structural disconnectedness, this non-attunement, is to be linked to the bodily make up of vertebrates at large, with the basic distinction between an internal body as a source of excitation and an external body as a motoric means of responding to this excitation in an attempt to diminish it, (iii) that it is relevant to introduce here the Freudian distinction between understanding and judging, and to identify understanding with the articulated motor-patterns of the external body that aim at grasping (com-prehending), and the judging with a dynamics of the mark, in which it is indicated (marked) that something stays together as a Thing exactly to the extent that it is not understood, not grasped through adequate motor-patterns, (iv) that mental representations (signifiers), understood as phonemic motor packages, are inscribed into this bodily dynamics of non-attunement, which means that they are particular motor-forms attempting to grasp that from which they are initially and structurally disconnected (the Thing), (v) that this distinction between understanding and judging, combined with the idea that signifiers or mental representations are motor-patterns, provides us with a basis to identify processes of repetition (*Wiederholungszwang*) in terms of repeated attempts situated at the level of the marks, structurally disconnected from what is satisfying at the level of needs, (vi) that the initial helplessness of the infant, together with the subtlety of language, with its (small and flexibly recombinable) phonemic motor-packages offered by the other/the Other, is the means through which the categorical difference between humans and other vertebrates can be made clinically relevant, and finally, (vii) that the representational grasping movement corresponds to objectification, whereby the object expresses the formal readiness of the representational space, a readiness that can be, in secondary instance, filled up in various ways, but that, due to the initial non-attunement in which it is grounded, is structurally missing the Thing that it initially marked, leading to an endless compulsion to repeat, without which? There would be no humanity, no culture, no subjective life.

In sum, we have argued for the inscription of the dynamics of signifiers in the structural non-attunement that already exists between actions and needs in mammals, leading to the repetition of actions independently from their being useful or not. Our purpose thereby was not at all to diminish the specificity of the human condition as a speaking condition. On the contrary, our purpose was thereby to show that we are tempted, time and again, to interpret human behavior too quickly as guided by intentional, consciously guided principles and mechanisms. Signifier repetition is the basic human condition, not intentional behavior! That is what Lacan stresses over and again, linked to the nature and the functioning of the signifier. In this way, Lacan’s viewpoint operates, more explicitly than Freud’s, a categorical shift from the idea that man is or should be guided by what brings satisfaction to his needs, to the idea that man is driven to repeat what was structurally missed. In speaking of a “conceptual intrusion” in relation to the compulsion to repeat, Lacan focuses on what constitutes the mental as a specific kind of object. In this, he wishes to “ensure,” “*faire valoir*” Freud (Lacan, 1966–1967/2017, XIII, p. 280) in what he was eventually after – the subject of the unconscious – and that is exactly the mental apparatus with as a module the compulsion to repeat.

## Author Contributions

GVdV is the main author of this article. She launched the core-idea of applying Freud’s distinction between judging and understanding to the process whereby a signifier is for the first time accepted in a dynamics that was until then a homeostatic pleasure dynamics. The articulation of this idea happened on the basis of already well-advanced research in neuropsychoanalysis on the signifier and on jouissance by AB. She is then second main author. The collaborative work between both these authors was intense and the result can be called a common result. SD contributed with her doctoral research on jouissance and the compulsion to repeat, part of the research background that served as a basis for this reflective article.

## Conflict of Interest Statement

The authors declare that the research was conducted in the absence of any commercial or financial relationships that could be construed as a potential conflict of interest.

## Footnotes

1 See, for instance, the end of II, where Freud speaks of tendencies beyond the pleasure principle, “more primitive than it and independent of it” (Freud, 1920/1955, p. 17), or at the end of III, where he states that the compulsion to repeat is “more primitive, more elementary, more instinctual than the pleasure principle which it over-rides” (Freud, 1920/1955, p. 23). We note the unhappy translation of *triebhafter* into more instinctual – the translation of *Trieb* by drive would have been more accurate. Luckily, this is remediated in the new translation from the upcoming *Revised Standard Edition*, edited by Mark Solms. A critical and annotated version of *Beyond the Pleasure Principle* has meanwhile been published in *Psychoanalysis and History* (Freud, 1920/2015).

2 “Quand Freud introduit pour la première fois, dans son *Jenseits* à lui, l’*Au-delà du principe du plaisir*, le concept de répétition comme du forçage, *Zwang*, répétition, *Wiederholung* – cette répétition est forcée: *Wiederholungszwang* – quand il l’introduit pour donner son état définitif au statut du sujet de l’inconscient, mesure-t-on bien la portée de cette intrusion conceptuelle? Si elle s’appelle ‘au-delà du principe du plaisir,’ c’est précisément en ceci qu’elle rompt avec ce qui, jusque-là, lui donnait le module de la fonction psychique, à savoir cette homéostase qui fait écho à celle que nécessite la substance de l’organisme, qui la redouble et la répète et qui est celle que, dans l’appareil nerveux isolé comme tel, il définit par la loi de la moindre tension. Ce qu’introduit la *Wiederholungszwang* est nettement en contradiction avec cette loi primitive, celle qui s’était énoncée dans le principe du plaisir” (Lacan, 1966–1967/2017, séance XI, p. 134).

3 cf. Freud (1900/1953, *The Interpretation of Dreams*, p. 461) and Freud (1920/1955, *Beyond the Pleasure Principle*, II, pp. 14–17). The game concerns a young child (Freud’s grandson) that used to play with his toys in such a way that he makes them disappear out of his sight, pronouncing then the long sound *o-o-o*. One day, while lying in his cradle, the child plays with his a bobbin, throwing it over the edge, pronouncing *o-o-o* again, and then, pulling the bobbin back in his little bed, triumphantly greets it with *a-a-a*. The two sounds are interpreted by Freud as expressing respectively *fort* and *da*, away and back.

4 That corresponds to the function of the ego. The ego cannot function but as a set of ‘permanently activated neurons,’ i.e., neurons that can retain excitation “Thus the ego is to be defined as the totality of the ψ cathexes, at a given time, in which a permanent component is distinguished from a changing one” (cf. Freud, 1895/1966, *Project for a Scientific Psychology*, p. 323).

5 That is why they can be said to be both operating in function of the death drive, as Freud explicitly admits in *Beyond the Pleasure Principle* (Freud, 1920/1955), VII, pp. 62 ff. In “The economic problem of masochism” (Freud, 1924/1962), however, Freud equates death drive with the Nirwanaprinciple, the pleasure principle and the reality principle as its modified form, with the representatives of libido.

6 Several comments are in order here. Firstly, there is one clear passage in *Jenseits* in which Freud speaks of the importance of the trait (the *Zug*) in relation to repetition: “This ‘perpetual recurrence of the same thing’ causes us no astonishment when it relates to *active* behavior on the part of the person concerned and when we can discern in him an essential character-trait (*Characterzug)* which always remains the same and which is compelled to find expression in a repetition of the same experiences.” We are much more impressed by cases in which the subject appears to have a *passive* experience, over which he has no influence, but in which he meets with a repetition of the same fatality (Freud, 1920/1955, *Beyond the Pleasure Principle*, II, p. 22, italics original). While, in the context of the discussion of *fort-da*, Freud stresses in the first place “the use of the object” and talks of pleasure stemming ‘from another source,’ Lacan explicitly interprets the “o-a” as signs or marks, in line with the passage we mentioned [for instance in *Logique du Fantasme* (Lacan, 1966–1967/2017), XI, p. 136 (italics original)], where he writes: “(…) identité significante du ‘plus’ ou du ‘moins’ comme signe de ce qui *doit* être répété [character-trait (…) which is compelled to find expression in a repetition],” and also in *The Four Fundamental Concepts* (Lacan, 1963–1964/1973), V, p. 54, where he discusses automaton and tuchè: “(…) l’insistance des signes à quoi nous nous voyons commandés par le principe du Plaisir.” That Lacan speaks of signs might be confusing, in as far as the sign here is purely formal and is defined in terms of oppositions. As such, it is what can be understood by the functioning of the signifier. Clearly, what Lacan intends is something different from more traditional (philosophical) accounts of the sign where it refers to something for someone (Peirce), or from cognitive views according to which the sign is what identifies stimuli on external grounds. With Lacan, following Freud, a sign or a mark is constituted by parameters or characteristics holding from within the subjective realm itself. As we explain further in this paper, Lacan speaks here of signs in reference to the *Einzige Zug*, which is the symbolic mark, the first signifier, that *indicates* that something was lost and cannot but be repeatedly searched for. By initiating that movement of repetition at the level of the signifiers, the little human being grafts himself upon the other, and inscribes himself in what Lacan calls *le champ de l’Autre*, the field of the Other (cf. Lacan, 1966–1967/2017, XVII, pp. 224–225/p. 136). Even if the distinction between the first and the second signifier, the *Einzige Zug* (the “sign”) and the “proper” signifier can only be made from within the realm of “significance,” the realm of the functioning of the signifier, it is nevertheless relevant to logically distinguish that first moment, the one in which something merely *indicates* that something was lost without being part of a differential system of signifiers. Of course, this is a mythical moment, set apart logically. Unless explicitly specified, we shall from here on speak of signifiers in order to avoid confusion between the philosophical use of the concept of sign and the psychoanalytical one. Secondly, to suggest that “another level” is initiated when dealing with signifiers, or, with Freud, to talk of pleasure as stemming from “another source,” is in agreement with the Freudian idea that memory and consciousness are two different and mutually exclusive systems: opening the possibility of using signifiers is opening the possibility of memory and immediately closes off their presence in consciousness. Thirdly, it is worth noting in passing that Freud speaks in the context of the use of signifiers, as in theater plays, of enjoyment, *Genuβ* (Freud, 1920/1955, p. 17) – the subject enjoys the commemoration of painful events in the play – a thing that will be of importance in the Lacanian viewpoint on enjoyment.

7 The use of terms such as “natural” is never unproblematic. In this context, we take it that Lacan intends natural as opposed to cultural. We do not want to open the philosophical discussion on nature/nurture here, but we do want to stress that the opposition is perhaps less straightforward than Lacan seems to suggest. We will argue further on that even if the compulsion to repeat is seen to be categorically distinguished from the pleasure principle grounded in a homeostatic dynamics, this does not mean that there are no biological constraints to be taken into account. As a matter of fact, what we intend to show is that the compulsion to repeat, even if it does not follow homeostatic principles, does have a logic that can be said to be biologically anchored and is clearly linked to precise biological constraints (Bazan and Detandt, 2013).

8 This is perfectly in line Freud’s “perpetual recurrence of the same thing,” stated in *Jenseits*, albeit not in the passage where we find *fort-da.* See footnote 6 for the full quote.

9 The term representation (as well as meaning, content, …) is extremely tricky. It is not the place here to unfold its various (mainly philosophical) traps and potentialities, but in relation to Lacan, it can be said that the representational realm mostly goes hand in hand with meaning, content, semantics, all of the imaginary order, against which he warns time and again. This is not *per se* the most adequate or the most interesting option though. We explain further on an alternative viewpoint on representation, one that is more radically embedded in the dynamics of the body, and that understands representations as *motor forms* that correspond to the central imagery that arises from action intentions that did not completely lead to discharge (see Jeannerod, 1994, p. 201; Bazan, 2007, pp. 125–126). The point we wish to make in relation to the above passage, is that in repetition, it is not the content that counts, but the *form*. As Lacan indicates, the child jumps toward the “o-a” in a movement of bridging what appeared as a lack in his vicinity. To interpret this “o-a” as a “representation” referring to the mother leaving the room, expected to come back through the door, adds too much and too quickly elements of content. What Lacan wishes to indicate, clearly in line with Freud – who also, in *Beyond the Pleasure Principle*, notes that the child is not in panic at the moment the mother leaves the room – is that it concerns a throw and pull-movement, i.e., a movement of the acting subject, doubled, over-written by a phoneme sequence, “o-a,” a signifier which is first and foremost a motor form. For more details (see Bazan and Van de Vijver, in preparation).

10 Here, is an example of how tricky the term representation can be. It is here used between inverted commas to highlight its traditional philosophical sense, i.e., something that stands for something for someone: the child fears that the mother, whom he saw leaving through the door, will perhaps not come back. Following our viewpoint on representation, and following also Lacan and Wallon in the interpretation of the case, we would say that the activated motor-pattern of the eyes while the mother left the room constitutes the representational structure, formal in nature, and that it is exactly that pattern that is repeated in the play with the bobbin and the accompanying “o-a”: the movement of throwing the bobbin away and pulling it back repeats the movement of something being at one point in the vicinity and at another point leaving a void in its absence.

11 In line with Aristotle, Lacan will add here that man not only thinks with his object, he *is*, as a subject, where the object is put into practice This probably refers to Aristotle’s (1984) idea that the mind is “none of the things existing in actuality before thinking” (*De Anima* iii 4, 429a24). In other words, thinking is nothing in actuality in abstraction of the form that thinks. Or still, our thinking is only with our objects of thought, that is, the forms. And that is precisely what is at stake here, and what we explain further on: “o-a” are formal objects that correspond to, or “stand for” actions tied together into motor packages that, in their opposite nature, organize what can be called subjective life.

12 Freudian pleasure, then, is not an affect in the common sense meaning of the word (namely, a hedonic or agreeable feeling). Freudian pleasure is relief, not delight. Therefore, it is not concerned with valence as affects are. Without going into details, we are inclined to see affect, in the common sense, as far less determining and orienting for behavior than the drive system and as not organized around the adequacy of the act (for more details, see Bazan and Detandt, 2013, 2015; Bazan et al., 2016; Detandt, 2016).

13 For a more extensive Lacanian discussion of the Thing, la Chose (see Lacan, 1959–1960/1986; Lew, 2014).

14 Our schema is also applicable to what happens in breathing, but it is different in the sense that the child, most commonly, finds by itself, through voluntary breathing movements, the adequate act that creates a relief of tension. In breathing – in contrast, e.g., with feeding – there is no constitutive need to for a contribution of the other, nor is there a difference in timing between finding the grasping movement (the breathing movement) on the one hand, and the satisfaction of the drive. We chose for the example of feeding, because it allows us to more straightforwardly articulate the different moments we wish to distinguish here.

15 This structurally missed step and the ways to retroactively recover it, whereby it is identified as the cause of our acting, is, actually, the ground for the hypothesis of the unconscious.

16 This event is, so we think, beyond the mechanical part of it, the explosion of the sugar receptors in the mouth massively and suddenly highly activated. As we have discussed elsewhere, a physiological marker is proposed for this surprise in the form of a release of dopamine at the level of the nucleus accumbens, i.e., the dopamine peak indicating unexpected reward (Bazan and Detandt, 2013) but also the dopamine release corresponding with disrupting, aversive and traumatic events (Bazan and Detandt, 2015), taken together as dopamine release ‘marking’ the unexpected event, independent of its valence (Bazan et al., 2016), and leading to a physiological registration known as ‘incentive sensitization.’

17 There can be a hesitation between *Triebrepräsentanz* and *Triebrepräsentant*, the first referring to the function of taking-the-place-of (“tenant-lieu”), the second to the taking-the-place-of itself. We wish to stress in the first place the notion of *Repräsentanz.* We shall see further on the delicate status of this point. For an extensive discussion of this concept (see Tort, 1966/2016), and for a subtle and pertinent “mise au point,” primarily in relation to the functional interpretation of the *Repräsentanz* (see Lew, 1983).

18 It would be possible and relevant to further elaborate on this issue in terms of alienation, as Lacan himself does all along in *Logique du Fantasme*. The subject (of the unconscious) is in “the part that is lost”; it is the subject of “je ne pense pas,” and that part is what shows itself “by surprise.” In relation to surprise, Lacan refers to Theodor Reik as the sole analyst having stressed its importance in relation to the unconscious (Reik, 1935/1976; cf. Lacan, 1966–1967/2017, VII, p. 92, *Logique du Fantasme*).

19 It was for instance translated as “représentant représentatif” (Laplanche and Pontalis) or by Lacan as “tenant lieu de la représentation” (see Tort, 1966/2016; Lew, 1983, for a discussion).

20 It is certainly not a genetically identifiable moment, as it is a moment of historization, indicating the registration of the contingency of a subject’s history in the form of a marked event. We do however think (as indicated in footnote 16) that there is a physiological correlate to this marking, in the form of a dopamine release, probably a dopamine spike. Note that if biological correlates can be situated at precise moments, their mental realization is dynamic, hence the use of the term ‘logical moment’ instead of ‘chronological moment’ (see also Bazan and Detandt, 2017).

21 See footnote 16.

22 Here, we easily jump from representations to signifiers. However, we propose that both are logically equivalent. Indeed, a representation (see footnote 7) is thought as a motor activation rest of an action intention that could not be discharged in actual motor activation; a signifier, then, is merely the application of this logic to the act of speaking, i.e., to articulatory phoneme motor patterns. Both representations in general, and signifiers specifically, are thus motor potentialities, i.e., forms, without any determined content or meaning.

23 See here Lacan’s discussion of the children’s play with “o-a.” To Lacan, from the moment the child uses “o” (S1), we cannot but add to it the differential “a” (S2), revealing the moment of S1 in isolation as a mythical, logical moment. He calls therefore the S1 symbolic, and states that there is from thereon no grounding to be looked for in the similarities or differences between objects such as toys, bobbins, mothers, to identify what a mark is. Nothing of this sort is hidden in the plays of Freud’s grandson that would *justify* the use of “o-a,” as nothing of this sort would *justify* the marked surprise effect in the child. According to Lacan, a unitary trait identifies something – in our interpretation, an event – but it is only through the repetition, in the differential play with other signifiers, that an event genuinely becomes a subjective event.

24 The first events in life frequently lead to the repetition of actions crucial for our survival, but our intuition is that this is at the bottom of it a matter of chance, not of teleology. E.g., a pigeon that made a wing movement before receiving a grain (Skinner, 1948), will from then on also repeat that wing movement because it had been registered as what had to be repeated, even if it had nothing to do with its survival chance (https://www.youtube.com/watch?v=8uPmeWiFTIw).

25 This is an ironic reference to Russell’s (1905) “On Denoting,” where he says, albeit from a different angle, but with, in our view, the same stakes at play, that there is no backward road from denotations to meanings.

26 The difference between *Repräsentanz* and *Repräsentant* can once more be brought forward here: however frozen or crystallized the motor pattern is (*Repräsentant*) it is nevertheless a motor-pattern, hence formal, potentially ready to receive different contents, and thus it is also functional in nature (*Repräsentanz*).

27 The resemblance with Kantian epistemology is straightforward. Kant’s dictum that “the Thing in itself is not knowable” serves as the starting point for his epistemology: from the moment we talk about objects and objectivity, we talk about what there is “for us,” in our words, what is within the range of the graspable through our motor patterns. Another way of saying the same thing, from within the formalistic tradition in philosophy (Frege in the first place) is that the grasping space is a functional space – in line with Kant’s philosophy that articulates the functionalism of Reason. It is from within the functional space that the place is prepared, delineated, circumvented, of what *can* come to satisfy the function. That place within a functional space, that is the object. In our words: that motor pattern ready to grasp something, that is the object. The formal discussion that is relevant in this regard, is the one on the relation between intension (that defines the function) and extension (that satisfies the function). Lacan works with these distinctions frequently, not in the least in his *Seminar XII*, *Problèmes Cruciaux pour la Psychanalyse* (Lacan, 1964–1965/2003).

28 There is no way, dixit Lacan, to conceive of an object without the dimension of satisfaction. With regard to the homeostatic, organic account, he says: “Rien, dans tout cela, qui pousse à la recherche, à la saisie, à la constitution d’un objet. Le problème de l’objet comme tel est laissé intact par toute cette conception organique d’un appareil homéostatique. Il est très étonnant qu’on n’en ait pas jusqu’ici marqué la faille. Freud ici, assurément, a le mérite de marquer, que la recherche de l’objet est quelque chose qui n’est concevable qu’à introduire la dimension de la satisfaction” (Lacan, 1966–1967/2017, p. 156).

29 It is even questionable whether we can speak of objects of satisfaction; the term “experience of satisfaction” seems more adequate, even if it also demands to be further unfolded, certainly in light of the meaning of the term “experience” in the philosophical tradition.
